# Name changes for fungi of medical importance, 2022–2024

**DOI:** 10.1128/jcm.02041-24

**Published:** 2025-09-03

**Authors:** Andrew M. Borman, Alireza Abdolrasouli, Elizabeth M. Johnson

**Affiliations:** 1 UK National Mycology Reference Laboratory, United Kingdom Health Security Agency South-West 436286 , Bristol, United Kingdom; 2 Medical Research Council Centre for Medical Mycology (MRC CMM), University of Exeter 601337, Exeter, United Kingdom; Vanderbilt University Medical Center, Nashville, Tennessee, USA

**Keywords:** taxonomy, classification, revisions, fungi, names

## Abstract

The current article summarizes the changes in nomenclature for fungi of medical importance published in the years 2022–2024, including new species and genera and revised names for existing ones. Most of the revised names have been widely adopted without further discussion. However, those that concern common pathogens of humans may take longer to achieve general usage, with new and current names reported together to engender increasing familiarity with the correct taxonomic classification.

## INTRODUCTION

Since fungal identification methods based on examination of morphological and phenotypic features are complicated by the astonishing diversity of organisms capable of causing human infections especially in immunocompromised hosts (1–5), molecular approaches to fungal identification have become de rigueur. However, molecular analyses have driven fundamental changes in fungal nomenclature and taxonomy as correct taxonomic relationships, and affiliations are elucidated: many phyla recognized as polyphyletic have been disbanded or substantially revised, cryptic species have been described in numerous classical morpho-species, long-extant species have been moved to new genera, and new taxa have been erected to accommodate novel organisms delineated by modern polyphasic phylogenetic analyses.

The above upheavals have been further compounded by the implementation of the Amsterdam Declaration (6) which, since 1 January 2013, prohibits the employment of separate names for the teleomorph (sexual) and anamorph (asexual) states of fungi, forcing mycologists to choose a single name (often from many alternative existing ones) for innumerable species. The practice of assigning precedence to the teleomorph name over anamorph alternative(s) was also abandoned in this amended International Code of Nomenclature for algae, fungi, and plants (ICN), thus allowing any of the multiple published legitimate names for a given species to be proposed as the correct name. To lessen nomenclatural instability, working groups and committees have been established under the auspices of the International Commission on the Taxonomy of Fungi (ICTF) and the Nomenclature Committee for Fungi (NCF) to propose lists of retained (protected) and rejected names for key species/genera, with only definitive changes being ratified. More recently, large international working groups have been established, endorsed by numerous learned societies, to attempt to develop consensus guidelines for fungal name changes (7) and standardize fungal disease terminology (8) so that nomenclatural changes do not unnecessarily impact medical mycology and clinical practice.

Currently, there is no single source available that records all nomenclatural changes proposed for fungi of medical importance. Novel fungal taxa and proposals to reassign or rename existing taxa are published continually in an ever-increasing range of scientific journals. For new names and combinations to be accepted as validly published, the ICN requires that all such taxa are registered in recognized online repositories. The principal repositories, Mycobank (http://www.mycobank.org/) and Index Fungorum (https://www.indexfungorum.org/), are, thus, invaluable sources of up-to-date taxonomic information. However, given the speed of change, even they are not complete/correct across all genera of medically important fungi. The current article represents an update to four previous ones (9–13) which provided lists of novel taxa and revised names for existing taxa for fungi of medical importance published between 2012–2015 (9), 2016–2017 (10), 2018–2019 (11, 12), and 2020–2021 (13). A number of overarching reviews that summarize recent changes, explain their mycological rationale, and propose approaches for seamless transition in the laboratory are also available (14–16).

## MATERIALS AND METHODS

To capture new fungal taxa and nomenclatural revisions described between 2022 and 2024, systematic literature searches were conducted in the PubMed database (https://pubmed.ncbi.nlm.nih.gov/) using the following search terms: “fungal sp. nov.,” “fungal gen. nov.,” “sp. nov. mycosis,” “fungal new species,” “fungal new genus,” “novel fungus,” “ascomycete sp. nov.,” “basidiomycete sp. nov,” “mucorales sp. nov,” “fungal taxonomic revision,” and “fungal comb. nov.” In addition, Mycobank and Index Fungorum were extensively searched to find taxonomic changes and additions to the most common fungal genera associated with human disease. The last date of access to these resources was 15th May 2025.

The novel taxa retained for inclusion here were those that had been recovered from human specimens; in some instances, a proven etiological role in human infection has been established; in others, the clinical significance of the organism remains unknown. New species/genera from veterinary sources were excluded from our lists, even if they were proven agents of infection, and likely to be causes of human mycoses in the future. Similarly, extant species which had been recognized as agents of human disease for the first time have been excluded here. As in previous iterations of this review (9–13), unless otherwise stated, the names listed in Tables 1 and 2 of the current article are those that fulfill the ICN rules for valid publication in that they (i) are in Latin binomial form, (ii) are accompanied by a description in Latin or English, (iii) have a holotype deposited in a recognized culture collection, and (iv) have been registered in MycoBank and published with a MycoBank registration number.

**TABLE 1:** List of new fungal taxa from clinical material for the period 2022–2024^*a*^

Species	Order	Source(s)	Clinical relevance	Reference	MB^*e*^ accession no.
*Aspergillus hubkae*	*Eurotiales*	Bronchoalveolar lavage	Aspergillosis	17	MB 850133
*Aspergillus pseudoalabamensis*	*Eurotiales*	Tracheal aspirate, sinus, ear	Aspergillosis	18	MB 847407
*Aspergillus neotritici*	*Eurotiales*	Toes nails, abdominal cavity	Not established	19	MB 844204
*Candida khanbhai*	*Saccharomycetales*	Blood, skin	Fungemia	20	MB 846114
*Candida massiliensis*	*Saccharomycetales*	Sputum	Not established	21	MB 845931
*Clavispora sputi*	*Saccharomycetales*	Sputum	Not established	22	MB 571973
***Inopinatus corneliae***	*Onygenales*	Skin	Not established	23	MB 854687
*Malassezia polysorbatinonusa^b^*	*Malasseziales*	Skin	*Not established* ^*c*^	24	MB 854169
*Mucor germinans*	*Mucorales*	Sputum	Not established	25	MB 853494
*Phaeoacremonium indicum*	*Togniniales*	Knee abscess	Phaeohyphomycosis	26	MB 850314
*Simplicillium sinense*	*Hypocreales*	Skin scrapings	Tinea facei*^d^*	27	MB 847412
*Syncephalastrum massiliense*	*Mucorales*	Sputum	Not established	28	MB 843858
*Syncephalastrum timoneanum*	*Mucorales*	Nail	Not established	28	MB 843870
***Tardiomyces depauwii***	*Saccharomycetales*	Blood, sputum, urine	Fungemia	29	MB 853439*^f^*
*Trichosporon austroamericanum*	*Trichosporonales*	Trichosporonales	Fungemia	30	MB 850273

^*a*^ Entries in bold denote novel genera.

^*b*^ Published under the name *Malassezia polysorbatinonusus*, orthographic variant.

^*c*^ Isolated from a patient with seborrheic dermatitis, but causal link not proven.

^*d*^ Repeat isolation not attempted; but fungal elements were seen microscopically.

^*e*^ MB, MycoBank.

^*f*^ Invalid: the genus *Tardiomyces *was invalidly described (published description did not include the MycoBank number).

**TABLE 2:** List of revised fungal taxa from 2022 through 2024^*a*^

Previous species name(s)	Revised species name	Order	Reference	MB accession no.
*Candida allociferrii*	*Blastobotrys allociferrii*	*Saccharomycetales*	31	MB 844756
*Candida auris*	***Candidozyma auris***	*Saccharomycetales*	32	MB 848169
*Candida blankii*	***Tardiomyces blankii***	*Saccharomycetales*	29	MB 853435^*b*^
*Candida digboiensis*	***Tardiomyces digboiensis***	*Saccharomycetales*	29	MB 853436^*b*^
*Candida duobushaemulonii*	***Candidozyma duobushaemuli***	*Saccharomycetales*	32	MB 848171
*Candida ethanolica*	*Pichia ethanolica*	*Saccharomycetales*	33	MB 571641
*Candida haemulonii*	***Candidozyma haemuli***	*Saccharomycetales*	32	MB 848173
*Candida inconspicua*	*Pichia inconspicua*	*Saccharomycetales*	33	MB 571647
*Candida intermedia*	***Sungouiella intermedia***	*Saccharomycetales*	32	MB 848202
*Candida khanbhai*	***Candidozyma khanbhai***	*Saccharomycetales*	32	MB 848176
*Candida melibiosica*	***Helenozyma melibiosica***	*Saccharomycetales*	32	MB 852178
*Candida pseudohaemulonii*	***Candidozyma pseudohaemuli***	*Saccharomycetales*	32	MB 848180
*Candida pseudointermedia*	***Sungouiella pseudointermedia***	*Saccharomycetales*	32	MB 848203
*Candida vulturna*	***Candidozyma vulturna***	*Saccharomycetales*	32	MB 848182
*Dipodascus/Galactomyces geotrichum*	*Geotrichum galactomycetum*	*Dipodascales*	34	MB 846379
*Lacazia loboi*	*Paracoccidioides lobogeorgii*	*Onygenales*	35	MB844093
*Saprochaete capitata*	*Magnusiomyces capitatus*	*Dipodascales*	34	MB 500138
*Saprochaete clavata*	*Magnusiomyces clavata*	*Dipodascales*	34	MB 554295
*Trichophyton indotineae*	*Trichophyton mentagrophytes var. indotineae*	*Onygenales*	36	MB 847789

^*a*^ Entries in bold denote novel genera.

^*b*^ The genus *Tardiomyces *was invalidly described (description did not include the MycoBank number), rendering all species within the genus also invalid.

## RESULTS AND DISCUSSION

The novel fungal taxa from human samples described between 2022 and 2024 are presented in Table 1 and include new (often cryptic) species in a number of established major genera of human pathogenic fungi including *Aspergillus*, *Candida, Clavispora, Malassezia, Mucor, Syncephalastrum,* and *Trichosporon*. For the first time in over a decade of updates, Table 1 does not include any novel dermatophyte species. *Inopinatus corneliae* sp. nov. gen. nov. (*Onygenales*), which was isolated from human skin and shown to be keratinophilic *in vitro,* is potentially the closest to a novel dermatophyte relative although no proven role in human disease accompanied its description (23). In fact, of the 16 novel fungal taxa associated with human clinical samples listed in Table 1, clinical relevance was established for only six. These included two novel, cryptic species of *Aspergillus*: *A. hubkae* (Section *Nigri;* [17]) and *A. pseudoalabamensis* (Section *Terrei;* [18]), associated with cases of invasive pulmonary and sinus infection, respectively (although additional isolates of *A. pseudoalabamensis* were also recovered from ear swabs and sputum); two ascomycetous yeasts from cases of fungemia: *Tardiomyces depauwii* sp.nov.gen.nov (29). and *Candida khanbhai,* a member of the *Candida haemulonii* complex and relative of *Candida auris* (20); a novel basidiomycete yeast in the genus *Trichosporon*, *T. austroamericanum,* which is an under-recognized cause of human *Trichosporon* infections (30); a new addition to the genus *Phaeoacremonium, P. indicum*, from a case of phaeohyphomycosis (26). *Simplicillium sinense* sp. nov. was isolated from skin scrapings from a patient with skin lesions consistent with tinea facei, and although fungal elements were detected in scrapings upon direct microscopy, repeat isolation of the fungus was not attempted (27).

Additional novel taxa in Table 1 for which clinical relevance was not formally established include *Aspergillus neotritici* (three isolates from toe nails, one from an abdominal cavity; section *Candidi*; [19]); two ascomycetous yeasts isolated from respiratory secretions, *Candida massiliensis* (21) and *Clavispora sputi* (published under the name *Candida sputum*; [22]); *Malassezia polysorbatinonusa* from skin from a patient with seborrheic dermatitis (published under the name *Malassezia polysorbatinonusus;* [24]); three mucoralean fungi: *Syncephalastrum massiliense* and *S. timonenau*m (28) and *Mucor germinans* (25). Although a number of *Malassezia* species have been implicated in human skin conditions including pityriasis versicolor and seborrheic dermatitis/dandruff (the precise pathogenic mechanisms remain to be elucidated), they are ubiquitous components of the human mycobiome and isolation from a symptomatic patient does not prove causation (37). However, with pityriasis versicolor, the finding of yeast cells accompanied by short, unbranched hyphae on direct microscopy is diagnostic for *Malassezia furfur* infection. The descriptions for *Syncepahalsatrum massiliense* (from sputum) and *S. timoneanum* (from human nails) cited optimal growth at 25°C and no growth at 40°C, suggesting that they might be poorly adapted to human body temperatures, although this was not explicitly stated. The isolate of *Mucor germinans* was recovered from respiratory secretions in an immunocompromised patient, where it presented as yeast-like cells producing multilateral budding daughter cells. This thermal dimorphism, which is shared with known pathogenic members of the genus *Mucor* including *Mucor circinelloides*, coupled with the ability to grow at 37°C, indicates that *M. germinans* can likely cause invasive human infection even if not formally proven in the initial case report. From the above list, it should be noted that one of the two new genera, *Tardiomyces,* is invalidly published as the original description did not include a Mycobank number, thus rendering all species within the genus (*T. depauwii* [Table 1]; *T. blankii, T. digboiensis*; Table 2) currently invalid.

Table 2 lists those extant fungal pathogens that have undergone taxonomic revisions over the last 3 years. As in previous recent iterations of this update, the list was bolstered by genus-wide taxonomic revisions of common human fungal pathogens including *Aspergillus*, *Cryptococcus,* and *Fusarium*. In this update, yeast species previously classified in the polyphyletic genus *Candida* predominate. For the first time, an organism appears in both Tables 1 and 2 of the same update: *Candida khanbhai*, initially described as a novel species in the *C. haemulonii* complex in 2023 (20), was transferred with all other members of the complex including the emerging pathogen *C. auris*, into the novel genus *Candidozyma* in 2024 based on extensive phylogenetic and phylogenomic analyses of the wider *Metschnikowiaceae* (32). At the same time, the species epithets for the “*haemulonii*” complex were amended to reflect the Greek-derived origin and latinised as 2nd declension neuter nouns in the generative case to give *Candidozyma haemuli, C. pseudohaemuli,* and *C. duobushaemuli* (Table 2). This same study resulted in the transfers of *Candida melibiosica* and *Candida intermedia/C. pseudointermedia* into the novel genera *Helenozyma* and *Sungouiella,* respectively, with all three organisms retaining their original species epithets (32).

A phylogenetic study by Visagie and coworkers (31) extended the genus *Blastobotrys* with several new anamorphic yeast species, including *B. allociferrii* (previously *Candida allociferii*), with the additional suggestion that *Blastobotrys* should prevail over the older teleomorph genus *Trichomonascus*, which would result in a new combination of *B. ciferrii* (currently *Trichomonascus ciferrii*) if the proposal is ratified. A similar phylogenetic reappraisal of a number of relatively rare yeast species with relatives in the large genus *Pichia* resulted in the transfer of *Candida ethanolica* into that genus (as *Pichia ethanolica*) and the additional proposal that *Candida inconspicua* should be renamed *Pichia inconspicua* (33). The latter will require further discussion as it has been accepted for over a decade, on the basis of mating studies and previous phylogenetic approaches, that *Pichia cactophila* is the teleomorph of *Candida inconspicua* (38). The final yeasts affected by taxonomic reassignments were the transfer of *Candida blankii* and *C. digboiensis* into the currently invalid new genus *Tardiomyces* based on phylogenomic approaches that confirmed their very distant relationship to the core species in the genus *Candida* (29).

The taxonomy of the arthroconidial yeast-like anamorph genera *Geotrichum* and *Saprochaete* and the parallel teleomorph genera *Magnusiomyces*, *Dipodascus,* and *Galactomyces* was recently revised to comply with the “one fungus, one name” principle (34). Phylogenetic analyses agreed with previous studies and supported the existence of two parallel monophyletic groups containing (i) *Geotrichum*, *Dipodascus,* and *Galactomyces* and (ii) *Magnusiomyces* and *Saprochaete*. Based on the principle of priority, *Geotrichum* and *Magnusiomyces* were retained as the representative genera, resulting in the species that had previously masqueraded under the various successive names *Endomyces geotrichum*/*Dipodascus geotrichum*/*Galactomyces geotrichum* being renamed as *Geotrichum galactomycetum and Magnusiomyces capitatus* and *M. clavatus* being retained in preference to *Saprochaete capitata* and *S. clavata*, respectively (34).

In 2023, Vilela and colleagues (35) addressed the hitherto confusing taxonomy of species in the genus *Paracoccidioides*, where cultivable species associated with disseminated infections (human paracoccidioidomycosis) and uncultivable species causing skin disease (Jorge Lôbo’s disease in humans and a similar presentation in dolphins) were long considered separate genera (*Paracoccidioides* and *Lacazia*, respectively). Successful sequencing of fungal DNA from skin lesions of dolphins infected with the uncultivable form and the original human pathogen reported by Lôbo (39, 40) revealed two distinct species both with high homologies with cultivable *Paracoccidioides* species, leading to the initial descriptions of *Paracoccidioides ceti* and *P. loboi* for the dolphin and human pathogens, respectively (40). However, since the new combination *P. loboi* was later found to be an illegitimate homonym, it was corrected with the introduction of the alternative name *P. lobogeorgii* for the pathogen of Lôbo’s disease (Table 2; [35]). In the same publication (35), the authors validated the additional species *P. lutzii* and *P. americana* which had both been invalidly published due to nomenclatural errors accompanying their original descriptions (13, 35). They did not validate the additional invalidly described sister species *P. restrepoana* and *P. venezuelensis* (13, 40) as further phylogenetic analyses failed to separate them from *P. brasiliensis*, with which they are now proposed to be conspecific (35; reviewed in reference 41). In an attempt to encourage standardized disease terminology (8), paracoccidioiomycosis ceti and paracoccidioidomycosis lobogeorgi have been proposed for the uncultivable diseases in dolphins and humans, respectively, with abandonment of the now obsolete disease term lobomycosis (41), a proposal that was endorsed by a recent expert-led multinational collaborative working group that aims to standardize fungal disease nomenclature (8).

Finally, disputes continue as to the preferred taxonomic status of *Trichophyton mentagrophytes* genotype VIII/*T. indotineae*. This emerging dermatophyte, first described as a cause of recalcitrant tinea cruris/corporis on the Indian subcontinent (42) and which has now spread worldwide to become one of the principal causes of tinea corporis (43, 44), often exhibits resistance to terbinafine *in vitro,* and infections are roundly agreed to be clinically resistant to the usual dosing regimens of this first line antifungal agent (42–44). This emerging pathogen can be distinguished from other members of the *T. mentagrophytes*/*T. interdigitale* complex by ITS genotyping. Based on various clinical and mycological features including the aggressive nature of infections, association with patients with links to the Indian subcontinent and poor response to terbinafine, this organism was erected to species level (as *Trichophyton indotineae*) in 2020 (45), a decision that was widely embraced by mycologists and clinicians alike due to the importance of distinguishing this particular agent of dermatophytosis from related clonal organisms in the *T. mentagrophytes*/*T. interdigitale* complex (46–48). More recently, other authors have argued that *T. indotineae* should be regarded as a variety of *T. mentagrophytes* due to difficulties in accurately identifying the organism, lack of monophyly in *T. mentagrophytes* and *T. interdigitale*, and to avoid further splitting of the *T. mentagrophytes*/*T. interdigitale* complex (36, 49). However, a number of studies have demonstrated that *T. indotineae* can be unambiguously distinguished from other members of the *T. mentagrophytes*/*T. interdigitale* complex using various genetic loci and even phenotypic characters (43, 50) and arguments remain that it is clinically very useful to distinguish those members of the complex that have already or are in the process of adapting to an anthropophilic lifestyle (*T. interdigitale, T. indotineae, T. mentagrophytes* genotype VII [51–54]) from those that remain principally zoophilic (the rest of the *T. mentagrophytes* complex). Although it is not the role of this update to arbitrate on taxonomy squabbles, these authors would support the continuous use of *T. indotineae,* especially considering the increasing clinical significance of this organism globally (42–47) and would also argue the importance of campaigns to raise awareness of clinicians and laboratorians alike to both of these emerging dermatophytes (*T. indotineae* and *T. mentagrophytes* genotype VII).

## CONCLUSION

As in previous editions of this update, we have endeavored to ensure that this review has captured most, if not all, of the proposed new or revised species names and nomenclatural changes affecting fungi of medical importance during the period 2022–2024. However, the list of novel taxa detailed in Table 1 is considerably shorter than in previous updates. As in previous editions, the novel species listed in Table 1 includes a mixture of newly recognized cryptic or sibling species in well-established taxa together with a smaller number of genuinely novel agents of superficial, subcutaneous, and disseminated human infections. Since some of these novel species have been described around a single isolate, and clinical significance has not been proven for the majority, understanding of their clinical relevance, prevalence, and whether the initial isolates are representative of the species will only be possible after examination of additional cases/examples. Likewise, determining whether cryptic species are sufficiently clinically different from their siblings to warrant identification beyond species complex level will require additional analyses (14).

The number of fungal taxa with proposed nomenclatural changes during the period 2022–2024 (Table 2) is similar in length to the lists published in earlier updates. Previous lists were inflated by taxonomic analyses spanning large genera and even families of clinically important fungi including the dermatophytes, genera within *Ajellomycetaceae,* various *Fusarium* species complexes, and pathogenic members of the genus *Cryptococcus* (9–13). Here, the majority (14/19) of the proposed changes concern yeast species previously classified in the polyphyletic genus *Candida,* with four new ascomycetous yeast genera described (*Candidozyma, Hellenozyma, Sungouiella,* and *Tardiomyces* [29, 32]) to accommodate ex-*Candida* species. One of these novel genera, *Tardiomyces,* requires validation (29). Of the 14 yeast species affected, the highest profile changes involved the reclassification of the emerging pathogen *Candida auris* (together with all other members of the *Candida haemulonii* complex) in the novel genus *Candidozyma* (32), with additional corrections of species epithets in the “*haemuloni* complex” to reflect their Greek origins.

*Candidozyma auris* has become globally notorious due to its propensity to drive large nosocomial outbreaks worldwide, almost universal resistance to fluconazole and the rapid emergence of multi-drug resistance during treatment (55–58). Given that in many geographic areas, *C. auris* is now one of the principal causes of invasive yeast infections (59, 60), it is perhaps surprising that its recent taxonomic revision has not to date received the same vocal public resistance seen when other ex-*Candida* species were subjected to equivalent changes (61–63). The lack of publications opposing this revision suggests that such vehement arguments against change are less pragmatic than claimed and may be driven by personal opinions concerning the perceived relative importance of different yeast species (63). However, we appreciate that using the metric of published opposition as set out in our statements above would not capture individual negative reactions to change at local and institutional levels. Additionally, it should be underscored that taxonomic changes that require erection of novel genera permit name changes to be relatively conservative (e.g., *Candidozyma auris* instead of *Candida auris;* [32]), a luxury that is not afforded in cases where a valid alternative name pre-exists: *Pichia kudriavz*evii, the revised name for *Candida krusei,* dates back to 1965 (11). Once again, we would reiterate that name changes that reflect biologically and clinically relevant differences in behaviors (including drug resistance, outbreak potential, etc.) are ultimately beneficial to patient care. The importance of using unambiguous names for fungi as a means of conveying important clinical and behavioral differences between apparently similar pathogens is also the reason that these authors support the continuous use of *Trichophyton indotineae* to describe the emerging dermatophyte previously referred to as *T. mentagrophytes* genotype VIII.  

Finally, although we advocate incorporating taxonomic updates into laboratory reporting wherever practicable, adopting new nomenclature is not mandatory as pointed out in the recent revised checklist item “MIC.11375 Review of Nomenclature” from College of American Pathologists Microbiology Committee. However, clinicians should at least be aware of alternative names for common organisms especially since certain of the multiple identification systems in use globally have already implemented changes. While it is ultimately the responsibility of individual laboratory directors to review updates and decide on future implementation, we would repeat our previous advice that changes can be managed, and unnecessary confusion avoided, by reporting new names alongside their previous ones until they have gained widespread recognition (11, 13).
